# Mediastinal Extrapleural Air Mimicking a Bulla in a Non‐Expandable Lung

**DOI:** 10.1002/rcr2.70693

**Published:** 2026-07-22

**Authors:** Hidenori Takahashi, Kota Taguchi, Mio Toyama‐Kousaka, Shinichiro Ota, Masaharu Shinkai

**Affiliations:** ^1^ Respiratory Disease Center Tokyo Shinagawa Hospital Tokyo Japan

**Keywords:** extrapleural air, mediastinum, pleuroparenchymal fibroelastosis, pneumothorax, unexpandable lung

## Abstract

In a patient with pleuroparenchymal fibroelastosis and a non‐expandable lung, CT and three‐dimensional rendering showed a bulla‐like mediastinal air pocket that regressed on follow‐up imaging and lacked an autopsy correlate, supporting intrathoracic extrapleural air rather than a true pulmonary bulla.

## Graphical abstract

A bulla‐like mediastinal air collection was observed in a patient with pleuroparenchymal fibroelastosis complicated by secondary pneumothorax and a non‐expandable lung. Internal web‐like strands and subsequent regression during drainage, together with the absence of an autopsy correlate, supported intrathoracic extrapleural air rather than a fixed pulmonary cystic lesion.

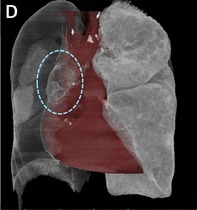

## 1 Clinical Image

A lifelong non‐smoking man in his 80s with pleuroparenchymal fibroelastosis developed secondary pneumothorax, predominantly on the right. Computed tomography (CT) with three‐dimensional volume rendering demonstrated a crescentic, bulla‐like mediastinal air pocket distinct from the adjacent lung, with internal linear strands resembling extrapleural fascial webs and a margin tracking the mediastinal pleural contour (Figure 1A–D). A chest tube was inserted and maintained for 3 weeks because repeated clamp trials failed despite cessation of the air leak within 1 day. During suction drainage and repeated pleurodesis, the lung did not re‐expand, and the water‐seal column rose progressively, suggesting increasingly negative pleural pressure. Serial CT scans obtained over the 3‐week drainage period showed progressive reduction of the mediastinal air pocket, with marked reduction on the final CT before discharge (Figure 2A–D). Three months later, CT obtained shortly before death showed no residual mediastinal air pocket, and autopsy revealed no bulla or cystic lesion at the corresponding site.

**FIGURE 1:**
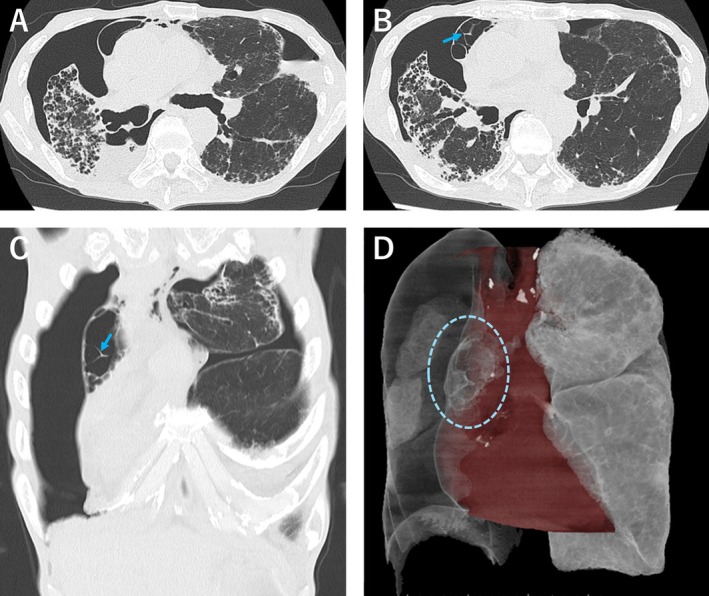
Initial computed tomography findings. (A–C) Axial computed tomography images (A, B) and a coronal multiplanar reconstruction (C) showing a right pneumothorax in the setting of pleuroparenchymal fibroelastosis and a crescentic, bulla‐like lucency adjacent to the mediastinum. Fine internal septation‐like linear strands resembling those of extrapleural fascial webs are present within the bulla‐like lucency on (B) and (C) (blue arrows). (D) Three‐dimensional volume rendering highlighting an anterior mediastinal air collection separate from the lung. The dashed blue circle indicates the air pocket along the anterior surface of the heart. The heart is shaded in red to provide an anatomical landmark.

**FIGURE 2:**
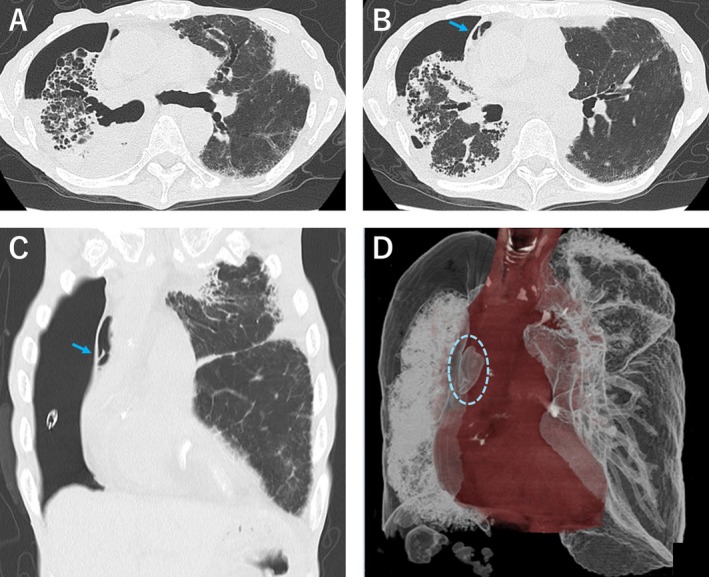
Follow‐up computed tomography obtained 3 weeks after admission during continued drainage. (A–C) Axial computed tomography images (A, B) and a coronal multiplanar reconstruction (C) showing a right pneumothorax with a reduction in size of the mediastinal bulla‐like lucency (blue circles). The internal septation‐like linear strands are still visible within the lucency. (D) Three‐dimensional volume rendering showing a decrease in size of the anterior mediastinal air pocket compared with the initial CT (dashed circle). The heart is shaded in red to provide an anatomical landmark.

The combination of absent parenchymal continuity on CT, no pathological correlate at autopsy, internal linear webs and reduction during drainage suggests that the mediastinal air pocket represented intrathoracic extrapleural air in a non‐expandable lung rather than a true bulla [1, 2].

## Author Contributions

H.T. and K.T. made substantial contributions to the conception of the work, conducted the clinical investigation and literature review, acquired and interpreted the data, and drafted the original manuscript, including the visualization. M.T.‐K., S.O. and M.S. contributed significantly to the data interpretation and critically revised the manuscript for important intellectual content. All authors have approved the submitted version and agreed to be accountable for all aspects of this study.

## Funding

The authors have nothing to report.

## Ethics Statement

All procedures were performed in compliance with relevant laws and institutional guidelines. Ethical approval for this case report was obtained from the Ethics Committee of Tokyo Shinagawa Hospital (Approval No. 24‐C11).

## Consent

The authors declare that written informed consent was obtained for the publication of this manuscript and accompanying images and attest that the form used to obtain consent from the patient(s) complies with the Journal requirements as outlined in the author guidelines.

## Conflicts of Interest

The authors declare no conflicts of interest.

## Data Availability

The data that support the findings of this study are available on request from the corresponding author. The data are not publicly available due to privacy or ethical restrictions.
